# Our Profession’s Loss

**Published:** 1899-08

**Authors:** 


### OUR PROFESSION'S LOSS.

THE death of Dr. Michael J. Treacy is more than the loss of a single member of the profession. He filled more than the average place of a leader in his calling. He was the leading spirit, the untiring devotee, the determined aggressor in urging army recognition of the veterinarian. He loved his work in the army, and died a martyr to its exactions. He gained the respect and good-will of every officer under whom he served by his faithful and intelligent discharge of duty. He demonstrated his own worth at every post, and thus taught the lesson that army rank was only the just due of his adopted calling. He never tired in the ofttimes hopeless struggle, but buoyed his colleagues by fresh sacrifices, renewed personal energy and earnest leadership, only to reach the edge of the goal he sought, but not to enjoy the fruits of his own labor. Dr. Treacy is dead, but the record of faithful work he has left will live long after him, and be a fruitful source of encouragement to those who may not have that same force of unrelenting efforts in the face of most discouraging conditions.

The army veterinarians have lost a wise leader, the profession a true and steadfast follower, and our country a faithful servant, without even the recognition or reward of the soldier in the ranks. How many more such instances does our country demand to be offered up as sacrifices, while it basks in ingratitude toward an honored and respected calling in every other country of the civilized world ?

